# More widespread alien tree species do not have larger impacts on regeneration of native tree species in a tropical forest reserve

**DOI:** 10.1002/ece3.6256

**Published:** 2020-04-12

**Authors:** Samson Aman Samson Kiswaga, John Richard Mbwambo, Deo Shirima, Ahmed S. Mndolwa, Urs Schaffner, René Eschen

**Affiliations:** ^1^ Department of Ecosystems and Conservation College of Forestry Wildlife and Tourism Sokoine University of Agriculture Morogoro Tanzania; ^2^ Tanzania Wildlife Authority Morogoro Tanzania; ^3^ Tanzania Forestry Research Institute Lushoto Tanzania; ^4^ CABI Delémont Switzerland

**Keywords:** Alien plant invasions, Amani Botanical Garden, direct and indirect effects, impact assessment, invasiveness

## Abstract

There is insufficient information regarding the factors affecting the environmental impacts of alien species. In particular, little is known about whether there is any relationship between the invasiveness (establishment and spread) of an introduced species and its per capita impact. We experimentally assessed the relationship between the extent of spread of up to 29 alien plant species and their impact on recruitment of native tree species in Amani Botanical Garden, Tanzania. We also studied the effects of allelochemicals of selected alien on native plant species to assess potential mechanisms of impact. We found no relationship between the extent of spread of an alien tree species and their impact on seed germination, seedling survival, and seedling communities of native trees in their understory, and no indication that allelochemicals consistently explain their effects on recruitment of the studied species. These results suggest that extent of spread cannot be used as a proxy for impact. Hence, managers should continue assessing both the spread and the impact of alien species when prioritizing alien species for management.

## Graphical abstract

Our manuscript describes the results from a combination of three studies with which we addressed the question whether there is a relationship between the spread of up to 29 alien plant species (trees, palms, and bamboos) and their impact on recruitment of native tree species. The results do not reveal a relationship, nor allow us to identify a consistent mechanism of impact of alien plant species on the recruitment of native tree species. We discuss the results in relation to published literature and the implication of our results for alien plant management decisions.

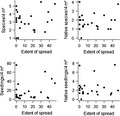

## 1 INTRODUCTION

A large number of woody plant species have been introduced, intentionally or unintentionally, into regions outside their native ranges (Richardson & Rejmánek, 2011). Intentional introductions of woody plants were done for a variety of reasons, for example, for ornamental purposes, for provisioning of firewood, or for afforestation. In many countries, commercial forestry and agroforestry operations are dominated by exotic tree species, which are widely used because of their presumed superior growth and other properties. Plant collections of botanic gardens often comprise many of such exotic species. Consequently, botanic gardens and (agro‐)forestry plantations in particular are important places of introduction that have led to the naturalization and invasion of alien plants (Guo et al., 2019; Hulme, 2011; Richardson & Rejmánek, 2011).

While originally the term “invasive alien species” referred to species that establish in a region outside their native range, spread, and cause environmental and/or socio‐economic impacts, more recent definitions focused on the alien organisms’ potential to establish and spread (Blackburn et al., 2011; Ricciardi & Cohen, 2007). Irrespective of the terminology to be used, it is essential to understand the factors affecting the spread of alien plant species as well as those contributing to the negative environmental and socio‐economic impacts. Following the conceptual approach of Parker, Simberloff, and Lonsdale (1999), the overall impact of an alien species can be defined as the product of the range size in the area of introduction, its average abundance per unit area across that range, and its effect per unit biomass or per capita on resident species. While the first and the second factors are likely to be linked to factors explaining the establishment and spread of alien species, the second and the third factors also relate to traits affecting the competitive interaction with resident species.

Increasing numbers of studies have documented how invasions by “transformer” alien plant species can negatively affect biodiversity and the ecosystem processes that are fundamental to human well‐being (Hulme, Burslem, et al., 2013; Pejchar & Mooney, 2009; Vilà et al., 2011). However, while considerable progress has been made in identifying species traits that increase the likelihood of successful establishment, naturalization, and spread of alien plant species (van Kleunen, Dawson, & Maurel, 2015), there remains a lack of predictors of an alien species’ impact on resident communities (Dick et al., 2017; Ricciardi, Hoopes, Marchetti, & Lockwood, 2013; Vilà et al., 2015). Intuitively, one may assume that alien species that have a higher establishment or naturalization success may also achieve higher densities and thus higher impact than species whose distribution is more restricted (Ricciardi & Cohen, 2007), but there is little evidence to support this. However, some alien species that are widely naturalized and spread rapidly appear to have little negative or even positive impacts on the environment (Hulme, Burslem, et al., 2013; Kueffer & Daehler, 2009). Vice versa, alien plant species may cause high negative impacts locally, but they spread only slowly (Ricciardi et al., 2013).

In a literature review, Ricciardi and Cohen (2007) did not find a relationship between the extent of spread and the extent of impact of alien invasive species of various organism groups based on a review of published studies. They noted, though, that most of the reviewed studies were based on correlations between the rate of spread of the invader and a decline in native species, rather than on experimental evidence. To our knowledge, experimental studies comparing the relationship between the extent of spread and the extent of impact of alien plant species are still lacking.

Most studies of the impact of alien plant species are limited to a few species and response variables and assessed impacts on a small spatial scale (Hulme, Pyšek, et al., 2013; Powell, Chase, & Knight, 2011). Yet, decisions about alien plant management, aimed at minimizing the risk to native biodiversity, require information about both actual impacts and potential impacts. For example, such information may be of importance for the management of reproducing alien species in botanic gardens, which have been the source of spread of the majority of the world's worst invasive plant species (Hulme, 2011) and which should target those species for management that have the largest impact on biodiversity and ecosystem functioning.

The impact of alien species on recruitment of resident plant species may be affected by the size of the per capita impact of the alien species and/or the local abundance of the invader. Alien plants may affect recruitment of native plants via mechanisms that have an impact on different life stages, such as seed germination, seedling emergence, and sapling growth. Mechanisms of impact may include changes in soil biotic and abiotic properties (Ehrenfeld, 2003; Ehrenfeld, Ravit, & Elgersma, 2005; Kuebbing, Classen, & Simberloff, 2014; Rajaonarimamy et al., 2017), allelochemicals (Hierro & Callaway, 2003; Murrell et al., 2011; Orr, Rudgers, & Clay, 2005; Weir, Park, & Vivanco, 2004), light availability and litter, which may affect light or allelochemicals (Augspurger, 1984a, 1984b; McAlpine, Howell, & Wotton, 2016; Muhanguzi, Obua, Oreym‐Origa, & Vetaas, 2005; Zhang et al., 2016), and apparent competition (Meiners, 2007). The impact of such mechanisms is variable and depends on site conditions (Ehrenfeld, 2003, 2010). Despite this contextual variation, a better understanding of the mechanisms underlying impact is important for both the identification of the invasive alien species that have the largest impact on native species and for their management (Levine et al., 2003). So far, only a few studies have directly compared the effects between invasive alien plant species (Hulme, Pyšek, et al., 2013), making it nearly impossible to generalize the effects of even a single mechanism. For example, a meta‐analysis of published studies to assess ecosystem impacts of alien plants found no significant difference between N fixing and other species (Vilà et al., 2011).

The overall objective of our study was to assess whether there is a relationship between the extent of spread of alien tree species from the original planting locations and their impact on the recruitment of native tree species in a botanic garden, which was set up in a protected area in tropical East Africa some 100 years ago. We assessed the effects of up to 29 alien plant species that differed in the extent of spread on germination, abundance, and diversity of native tree species. Specifically, we asked whether the spread of the planted alien plant species relates to (a) their per capita impact on germination, establishment, and survival of three native tree species and to (b) the abundance and species richness of seedlings growing in the understory of these alien species. We also studied possible mechanisms underlying the impact on native tree species, in particular the effect of allelochemistry.

## 2 METHODS

### 2.1 Study area

The study was conducted in the Amani Botanical Garden (ABG), which is part of the Amani Nature Reserve (ANR), located in the East Usambara Mountain range of Tanzania (5°06’01“S, 38°37’50”E). The area experiences heavy rainfall with mean annual rains of ca. 2,300 mm that are concentrated in March to May and October to November. The mean monthly temperature ranges from 16.3°C to 24.1°C. The forest vegetation is divided into lowland forest and submontane forests (Frontier Tanzania, 2001). The ABG was established in 1902 and is one of the oldest botanic gardens in Africa, with more than 1,000 species of plants from all over the world, many of which were planted within two decades following the establishment of ABG (Hulme, Burslem, et al., 2013). The ABG covers an area of 300 ha out of 8,380 ha of the total ANR area, which was gazetted in 1997 with an objective of protecting biodiversity and water catchments (United Republic of Tanzania, 1997). The forests in this reserve form part of a globally recognized biodiversity hotspot and are a center of high endemism and plant diversity (Hulme, Burslem, et al., 2013). The floristic composition of the region is very diverse: Approximately 276 species of trees (>10 m tall) have been recorded in the East Usambara Mountains and almost one fifth are likely to be endemic or near‐endemic (Rodgers & Homewood, 1982). Common native tree species in ABG include *Cephalosphaera usambarensis* (Warb.) Warb., *Allanblackia stuhlmannii* (Engl.) Engl., *Albizia gummifera* (J.F.Gmel.) C.A.Sm., *Beilschmiedia kweo* (Mildbr.) Robyns & R. Wilczek, *Diospyros abyssinica* (Hiern) F. White, *Englerodendron usambarense* Harms, and *Drypetes gerrardii* Hutch. Good records of the planting history of the plant collection in ABG exist, and a relatively recent inventory of the establishment and spread of some of the originally introduced plant species has been published by Dawson, Mndolwa, Burslem, and Hulme (2008).

### 2.2 Overall design of the studies

We addressed the objectives in three studies (Table 1). We assessed the effects of soil from under twelve alien plant species on the germination of three native species in a Petri dish experiment. The effects of twenty‐nine alien plant species on seedling emergence and establishment of the same native species, as well as on the abundance and diversity of saplings of native and alien woody plant species were assessed in two field studies.

**Table 1:** Summary of the objectives, methods, and key results of the three studies

Study	Main question	Method	Key result
1	What is the effect of soil from under twelve alien plant species on germination of three native species?	Germination of seeds of three native species in Petri dishes with soil collected under alien trees.	No effects of the extent of spread or the addition of activated carbon were found (Appendix S3).
2	What is the effect of alien plant species on seedling emergence and establishment of native tree species?	Germination of seeds of three native species planted at the base of alien trees in plots in Amani Botanical Garden.	The number of germinated seedlings was unaffected by the extent of spread (Figure 1).
3	Are the abundance and diversity of saplings of native and alien woody plant species affected by alien plant species?	Inventory of native and exotic seedlings at the base of alien trees in plots in Amani Botanical Garden.	Neither native or total seedling species richness nor abundance of native or all species showed a significant relationship with the extent of spread of the 26 alien plant species (Figure 2).

### 2.3 Native tree species

Three native tree species were selected for the manipulative studies in Petri dishes and in the field. The species were selected based on the availability of mature seeds at the time the studies were set up (mid‐February 2017). *Funtumia africana* (Benth.) Stapf is a medium‐sized pioneer species with small seeds crowned by beaks covered with long hairs that make them effectively dispersed by wind (Schulman, Junikka, Mndolwa, & Rajabu, 1998). It has a wide distribution in tropical Africa and is commonly found in moist light or secondary forests from sea level to 1,600 m.a.s.l. (Omino, 2002). Seeds can be stored up to 2 months but germinate best if sown immediately after collection (Omino, 2002). Although the tree usually does not form a wide crown, it casts heavy shade and its leaf litter is reported to improve soil fertility (Orwa, Mutua, Kindt, Jamnadass, & Simons, 2012). *Macaranga capensis* (Baill.) Sim is a medium‐sized pioneer species common in East Africa, Sudan, Ethiopia, Zimbabwe, Mozambique, to South Africa (Radcliffe‐Smith, 1987). Mature trees produce many small roundish seeds, with about 1,100 seeds per kg. It germinates profusely and can dominate in secondary forest gaps and edges between 300 and 3,000 m a. s. l. The seeds can be collected from the ground and sown without any pretreatment in the nursery or directly in the field (Msanga, 1998). *Isoberlinia scheffleri* (Harms) Greenway is a tall climax species with large seeds in large‐flattened woody pods. The diameter of mature seeds can reach up to 6 cm, and germination is promoted by abundant moisture (Msanga, 1998). The tree is only found in Tanzania, and it is common in moister parts of ANR above 450 m.a.s.l. (Schulman et al., 1998). Seeds of these species were collected in ABG immediately prior to the start of the studies. A sample of the seeds of each species was tested for seed viability before the start of the experiments, using the cutting test method of the Tanzania Tree Seeds Agency (TTSA) at Lushoto. Seeds were considered viable if the cotyledons were whitish, and seeds were considered unviable if the cotyledons were dark brown and somewhat rotten. Twenty seeds per species were inspected, and the large majority were viable (75, 100, and 85% of *M. capensis*, *F. Africana,* and *I. scheffleri*, respectively).

### 2.4 Alien plant species

To assess the relationship between the extent of spread of alien plant species and their ecological impact, germination and growth of native species was tested in the understory of, or in soil collected underneath a total of 29 alien species (Appendix S1). The alien trees and bamboos were planted in ABG at similar densities over a century ago. To ensure that the results were primarily affected by single plants (per capita effect), we sampled soil (Study 1) or conducted our studies (studies 2 and 3) near the base of individual trees, palms or bamboos, that is, always underneath the canopy of the selected plant. This approach has been repeatedly applied in studies assessing impacts of large plant species on heterospecific and conspecific seedling recruitment (e.g., Becerra et al., 2018; Chapman & Chapman, 1995; Packer & Clay, 2000; Reinhart, Packer, Putten, & Clay, 2003). The alien species were selected by a botanist with good knowledge of the history of species planted in ABG. Species from different plant families were included, and most families were represented by species with contrasting invasion history in ANR (see below). The selected alien species represent a range of growth forms, including trees, palms, and bamboos. Due to the topography and history of the ABG, the selected alien species were located at various altitudes and in different situations with respect to slope, exposition, soil type, and surrounding vegetation.

The extent of spread of each alien species was obtained from Dawson et al. (2008), who recorded the occurrence of each species in the number of plots of the ABG in addition to the original planting sites. The number was based on a survey of ca. 55% of the originally planted plots; the other plots were no longer existent. The number of additional plots thus is indicative of the extent of spread within the AGB, which likely reflects a combination of invasiveness and niche size of the species, but is not indicative of abundance in a plot. Although the majority of the hundreds of alien trees were planted over a period of ca. three decades (1902–1930; 117 of the species listed in Dawson, Burslem, and Hulme (2009) in the first decade and 23 after 1915), the long time since planting all of these species reduces the importance of the exact planting date of each species and differences in the extent of spread are unrelated to differences in the year of planting (RE, unpubl.). In addition to the exotic species, the three native species described above were also included in order to be able to compare the responses to alien species with those of native species. Because the extent of spread of the native species was unavailable and not relevant for our study, we assigned the mean extent of spread of all exotic species (excluding the very widely spread *Clidemia hirta*, thus 17) to these three native species. For Study 1 (see below), a subset of twelve alien species were selected, in such way that they represented different extents of spread and that species of the same family also represented contrasting extents of spread, while in studies 2 and 3 all species listed in Appendix S1 were used.

### 2.5 Study 1—Petri dish experiment

Soil for Study 1 and for analysis of the soil characteristics was taken from the top 20 cm at one, randomly selected location under four replicate trees of each species, at a distance of ca. 1 m from the trunk. Soil for analysis was stored in a fridge until use. Analysis of soil texture and chemical composition was carried out by the Mlingano laboratory in Muheza (Tanga District) according to standard laboratory protocols (Ministry of Agriculture & Fisheries, 2016). The mean values for the analyzed soil variables are given in Appendix S2. The texture of the soil in one of the 60 samples was not analyzed.

Petri dishes (10 cm diameter) were filled with soil taken from underneath individual trees. There were two Petri dishes with soil from each individual tree, resulting in eight replicates per soil origin. Four ml of activated carbon (Sigma Aldrich, St. Louis, MO) was added at a concentration of 20 ml/L to half of the Petri dishes to absorb organic compounds (Murrell et al., 2011). Petri dishes were watered and left for 24 hr prior to sowing of seeds to allow activated carbon to absorb soil allelochemicals. Petri dishes were arranged randomly on a laboratory table at room temperature at the Lushoto Silviculture Research Center (4°47’24“S, 38°17’43”E, ca. 1,350 m.a.s.l.). Three seeds each of *F. africana*, *M. capensis,* or one seed of *I. scheffleri* were sown per Petri dish, taking care that the seeds were covered by soil in order to avoid drying of the seeds and maximizing contact with the soil. There were 360 Petri dishes (15 species x 3 seed treatments x 8 replicates). Petri dishes were watered as needed, and the number of germinated seeds in each Petri dish was counted and recorded for 90 days from the date the experiment was set. The only species that germinated was *F. africana,* and the other two species were therefore excluded from the analyses.

### 2.6 Study 2—Germination and survival in the field

Seeds of *F. africana*, *M. capensis,* and *I. scheffleri* were sown under 29 alien and three native species in ABG in March 2017. Under one individual tree of each of the 32 species, ten seeds of each native species were sown in a randomly placed area (ca. 0.5 x 0.5 m per species) 1 m away from the trunk of the tree. Any leaf litter that was present was removed prior to sowing and put back afterward to mimic the original conditions under the alien species. The locations where seeds were sown were marked, and the number of seedlings was counted after two and eight months.

### 2.7 Study 3—Recruitment under alien plant species in ABG

In March 2017, the natural recruitment of woody species was recorded under alien and native plant species in ABG. Four 2 x 2 m quadrats were laid in the cardinal directions at ca. 1 m from the stem base of one randomly selected individual of each of 29 alien and three native tree species. In four cases (*C. robusta*, *L. camara*, *N. dullooa,* and *S. occidentalis*) seedling occurrence was assessed in fewer than four quadrats; these species were excluded from the analysis. In each quadrat, all seedlings of woody plant species with a height less than 1 m were identified to the species level and counted. The seedlings of native species were assigned to successional stages (pioneer, secondary, and climax) according to their growth characteristics and reproductive habit, based on Hamilton and Bensted‐Smith (1989) and de Moraes, Luchiari, Assumpcà, Puglia‐Neto, and Sampaio Pereira (2002) (Appendix S4). The litter depth was measured using a ruler and litter cover estimated visually.

### 2.8 Statistical analyses

The effect of the extent of spread of alien species and activated carbon on the number of germinated seeds in Study 1 (Petri dish experiment) was analyzed using a generalized linear mixed effects model with the number of germinated seeds as response variable, the extent of spread of the species under which soil was collected, the life form of those species, whether the plants were alien or native and activated carbon as fixed effects and a quasipoisson data distribution. The fifteen species were included as random factor. Differences in soil chemistry among the fifteen plant species were tested using individual linear mixed effects models with the soil chemical parameters as response variables, the extent of spread of the species under which soil was collected and altitude as fixed effects. The fifteen species were included as random factor. Relationships between the number of germinated *F. africana* seeds and individual soil chemical parameters were analyzed using generalized linear mixed effects models with the number of germinated seeds as response variable, the chemical parameters as fixed effects, and a quasipoisson data distribution. The fifteen species were included as random factor. *Clidemia hirta* was omitted from the analyses of studies 2 and 3, as the extent of spread was far greater than that of the other species and it had an outsize influence on the results. The data of Study 2 (field experiment) were analyzed using a generalized linear model with quasipoisson distribution because of the large number of instances where no seedlings were found. The number of germinated seedlings and surviving seedlings was response variables and the extent of spread of the 29 species under which the seeds were sown, species origin (native or alien), their life form, and the identity of the sown native tree species explanatory factors.

For analysis of the abundance and species richness of seedlings in Study 3 (the natural recruitment), seedling species were divided into native and alien. For each of the 25 alien and three native species under which the inventory was made, the average number of seedlings and the average number of species per 4 m^2^ were calculated, resulting in a single value for each response variable per tree species. General mixed effects model with altitude, the extent of spread, successional stage of the seedlings (alien, and native pioneer, secondary, or climax), the life form, and whether the trees were alien or native as explanatory variables and the tree species under which the assessments were carried out as random variables were used to explain differences in seedling number and the number of seedling species. Both models had assumed quasipoisson distributions. Differences in litter cover and litter depth were tested using generalized linear models with the extent of spread and the life form of the plant species under which the assessment was carried out and whether the plant species was alien or native as explanatory variables and an assumed quasipoisson distribution. Correspondence between seedling abundance and species richness was assessed using Pearson correlation. All statistical analyses were performed using R (Core Team, 2017). Mixed‐effects models were calculated using the glmmPQL function of the “MASS” package and ANOVA‐style summaries produced using the ANOVA function in the package “car.”

## 3 RESULTS

### 3.1 Soil characteristics

The characteristics of the soils differed among the fifteen tree species, but no significant effects of the extent of spread of the species were found (*p* > .05; Appendix S2, S3). The Ca, P, K, Cation Exchange Capacity, and pH were negatively and Na, organic carbon content, and percent clay positively related to altitude (*p* < .05; Appendix S3).

### 3.2 Study 1—Petri dish experiment

On average, one in three *F. africana* seeds in each Petri dish germinated (1.1 ± 0.1 SE). Differences in germination rate were not affected by the extent of spread of the alien tree species (*p* > .05; see Appendix S3 for full model outputs) or by activated carbon (*p* > .05). A positive relationship between the number of germinated *F. africana* seeds per Petri dish (with activated carbon) and organic carbon content of the soil was found (*p* = .003). No significant relationships with other soil characteristics were found.

### 3.3 Study 2—Germination and survival in the field

**Figure 1:**
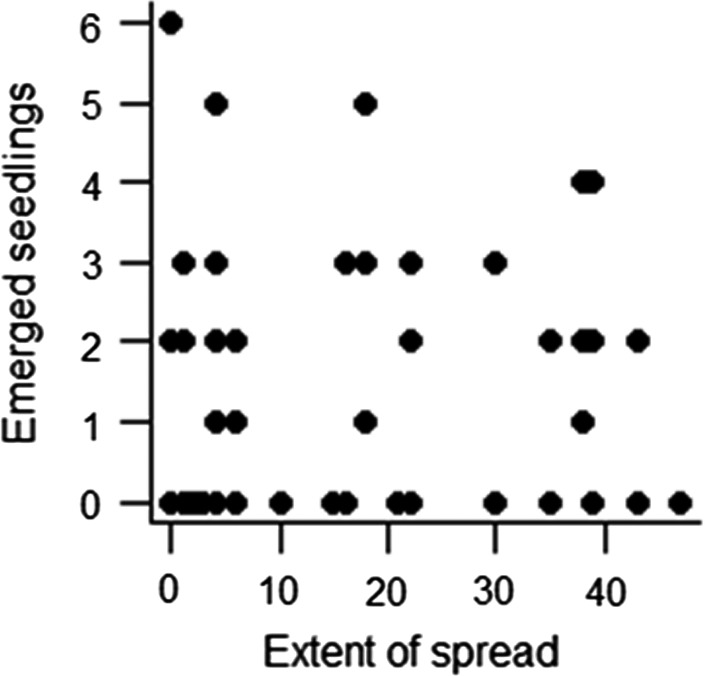
The number of seedlings of *Funtumia africana*, *Macaranga capensis,* and *Isoberlinia scheffleri* (combined) present after eight months. Seeds were sown under 28 alien plant species differing in the extent of spread in the Amani Botanical Garden (ABG). *Clidemia hirta* was omitted from the analysis. The extent of spread was quantified as the number of compartments in the ABG where a species was recorded outside its original planting location (Dawson et al., 2008)

Out of the 1,140 sown seeds, 82 emerged after two months and 19 survived eight months after sowing. More seedlings of *F. africana* than of the other two species were found (an average of 1.6 ± 0.3, 0.3 ± 0.1, and 0.3 ± 0.1 seedlings of *F. africana*, *I. scheffleri,* and *M. capensis* in each location, respectively; *p* < .001). No significant relationship between the number of germinated seedlings and the extent of spread was found (Figure 1). The average number of *F. africana* seedlings present after eight months was more than ten times higher than that of the other two species (*p* < .001), and there was no effect of the extent of spread of the 29 alien species on seedling survival (*p* > .05).

### 3.4 Study 3—Recruitment under alien plant species in ABG

Seedlings belonging to a total of 77 species were recorded, of which 27 were alien and 50 were native. The total number of alien seedlings was more than six times the number of native seedlings (3,744 versus 655). As a consequence, the average number of alien seedlings was more than ten times higher than the average number of native seedlings. The number of seedlings belonging to native pioneer and climax species was higher than that of secondary species (2.5 ± 0.6, 11.4 ± 2.6, and 10.3 ± 3.9 per 4 m^2^, respectively; *p* < .001; Appendix S3). More alien than native seedlings were found under alien trees, but no such difference existed under native tree species (Successional stage x Alien/native interaction; *p* < .001). This resulted in on average a higher number of alien seedlings than native seedlings of pioneer, secondary, or climax species (successional stage; *p* < .001).

**Figure 2:**
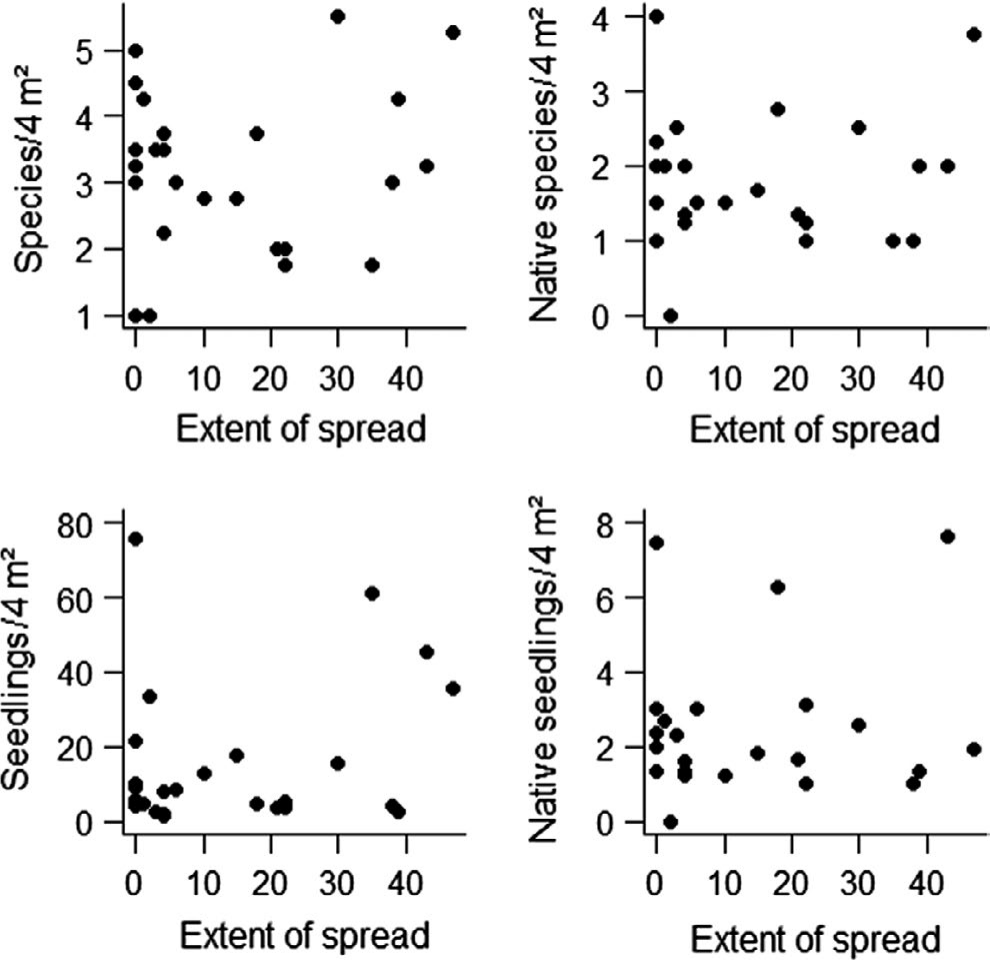
Relationship between the invasiveness of 25 alien plant species and species richness and abundance (top and bottom graphs), both for all species (left graphs) and native species only (right graphs), in the Amani Botanical Garden. None of the relationships was significant (all *p* > .2)

There was substantial variation in seedling species richness and abundance under trees of the 28 different plant species (average of 3.2 ± 0.2 species and 15.9 ± 3.4 seedlings per 4 m^2^). However, neither native, total seedling species richness nor abundance of native or all species showed a significant relationship with the extent of spread of the 25 alien plant species (all *p* > .05; Figure 2). There was a significant, but weak positive relationship between seedling species richness (*p* < .05).

A total of 187 seedlings of *F. africana* and 60 seedlings of *I. scheffleri* were found, but the average number of seedlings per quadrat was not related to the extent of spread of the alien tree species (both *p* > .05; 1.5 ± 0.6 and 0.5 ± 0.4 seedlings per 4 m^2^, respectively). No seedlings of *M. capensis* were found.

**Figure 3:**
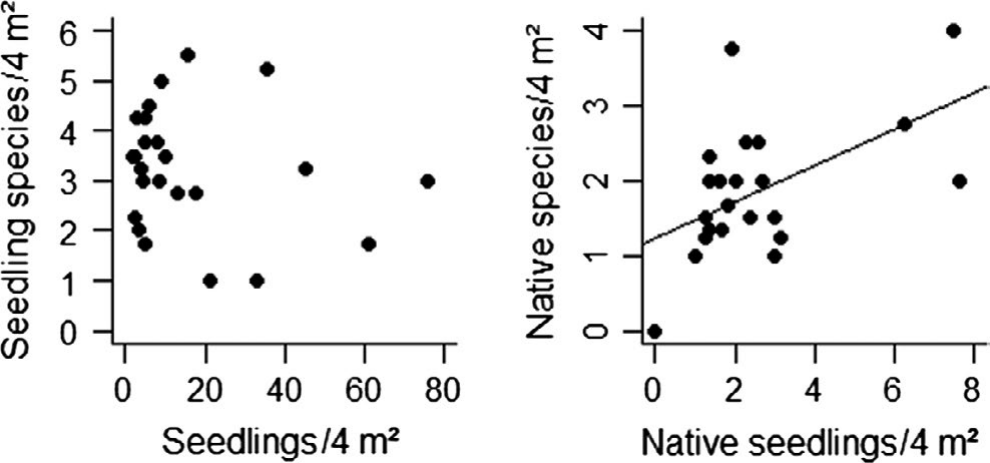
The relationship between seedling abundance and seedling species richness under 25 alien tree species in the Amani Botanical Garden. The left figure shows all recorded seedlings and the right figure only seedlings of native species

No significant correlation was found between total species richness and the total number of saplings (*t* = −0.815, *p* > .05; Figure 3). Also, there were no significant correlations between species richness of native and alien seedlings (*t* = 0.714, *p* > .05) or between the abundance of alien and native seedlings (*t* = 0.769, *p* > .05). A positive correlation between species richness and abundance of native (*t* = 3.0064 *p* = .007), but not of alien seedlings (*t*=−0.622, *p* > .05) was found.

There was variation in the litter depth and cover under the trees (average of 4.4 ± 0.6 cm of litter and 65 ± 5% litter cover), but these were not significantly affected by the extent of spread of the alien tree species (*p* > .05). The seedling abundance and species diversity, total, alien or native, were not affected by litter depth or cover (*p* > .05).

## 4 DISCUSSION

Our results revealed no evidence for a relationship between the extent of spread of alien plant species in ABG and their impact on germination, abundance, or diversity of native, alien, or all species together. These findings corroborate those of a literature review by Ricciardi and Cohen (2007), but our results are based on three separate experimental studies, with alien species that have a similar introduction history and that were introduced more or less at the same time. All tree species were planted in a protected area with relatively low amounts of habitat disturbance. Also, our study benefitted from the standardized approach taken in the Petri dish and field experiments that reduced other confounding effects such as seed availability and, in the case of the Petri dishes, climate. It is not clear what causes the interaction between altitude and successional stage in Study 3, but there seemed to be a positive relationship between climax species richness and altitude and the opposite for pioneer species, whereas no clear relationship between species richness and altitude was found for alien species and secondary successional species.

Species‐specific differences in responses to alien species, in particular the responses of pioneer and late successional species, may occur, but this has not been addressed in previous studies (Hulme, Burslem, et al., 2013). In our experimental studies, we worked with tree species belonging to different stages of succession, but the germination rate of *M. capensis* and *I. scheffleri* was very low, which, in addition to the small number of species sown, makes it impossible to generalize the results. It is unclear why the germination rate of the untreated seeds was low, but *M. capensis* seeds are eaten by birds (Dowsett‐Lemaire, 1988) and passage of the gut may stimulate germination, while *I. schefflera* seeds swell and produce mucus when soaked in water, which results in very rapid germination (RE, pers. obs.), and the experimental conditions in the Petri dish study may have been suboptimal for these species. However, the seedlings of 50 native species recorded under alien trees in the ABG allowed us to make this comparison and the results indicate that there was no difference in the effect of the extent of spread of alien plant species on seedling establishment among native tree species of different successional stages. This may be due to a lack of a single mechanism of impact of the alien species on the diversity of native species recorded in the field study, or, if there was a single mechanism this may not have affected spread of the alien species. The germination and establishment of the latter may also be affected by a variety of environmental factors, such as allelochemicals, nutrients, and light (Augspurger, 1984a; Stinson et al., 2006).

The relationship between spread and impact of alien plants has rarely been studied (Levine et al., 2003; Pyšek et al., 2012; Ricciardi & Cohen, 2007) and, as far as we are aware, our study is the first to test this relationship experimentally. Our study did not reveal patterns in the impact of alien species on recruitment of native tree species in ABG, nor did they provide evidence for the explanations for potential differences in impact among the studied alien plant species. In particular, the results of our study suggest no consistent effect of allelochemicals on germination across the alien species tested. The only indication for a mechanism came from the positive relationship between soil organic carbon content and germination of *F. africana* in Petri dishes, and we suggest that higher carbon, and thus organic matter content, increased soil moisture content, which in turn promoted germination of *F. africana*. However, organic carbon content was not related to the extent of spread of the alien plant species. Only a few potential mechanisms of impact were assessed in this study, and we focused on impacts on germination or emergence. Mechanisms of impact vary depending on life stage of the plant. For example, seed germination may be affected by the soil, either through allelochemicals or abiotic factors, or by light (Hierro & Callaway, 2003; Khurana & Singh, 2001; Kyereh, Swaine, & Thompson, 1999). Seedling establishment may be affected by biotic and abiotic factors, as well as predators (Augspurger, 1984a; Stinson et al., 2006). Finally, early growth and survival may be affected by light and soil biotic and abiotic factors, including plant–soil feedback (Battaglia, Fore, & Sharitz, 2000; Mangan et al., 2010).

It is unlikely that the changes in ecosystem properties caused by alien species, and thus the mechanisms of impact such as changes in soil nutrient content, are qualitatively or consistently different from native species impacts on ecosystem processes (Ehrenfeld, 2010). This is illustrated by the variation in characteristics of the soil taken from under alien plant species, as well as in germination of the native species in those soils, which did not deviate significantly from the range of the three native species that we studied as reference. The differences in the effects of alien plant species on naïve or experienced neighboring plants have been explained by interactions mediated by allelochemicals (Hierro & Callaway, 2003). Adding activated carbon is a method that has been used to study the effects of allelochemicals in alien plant species research (e.g., Murrell et al., 2011). However, activated carbon can affect the availability of beneficial root exudates and some soil nutrients (Weißhuhn & Prati, 2009); activated carbon can represent a source of phosphorous (Lau et al., 2008), but the added amount would have been the same for all Petri dishes in our study and we did not find a relationship between germination rate and phosphorous content of the soil. The only soil effect on germination of *I. scheffleri* appears to have been related to the organic matter content of the soil, which stimulated germination of the very large seeds of this species. Hence, our study did not provide evidence that allelochemicals play a consistent role in the impact of alien species on germination and seedling establishment of native tree species.

Often it is unclear whether introduced species are drivers of impacts on native species, or whether they are “passengers of change” (Macdougall & Turkington, 2005), whose establishment in locations with favorable conditions coincides with changes in native species abundance. The alien species we studied were planted intentionally in the plots where our studies were carried out. The studied trees have been present at the planting locations for over a century and were planted in a range of environmental conditions. The ABG is a protected area that has been little disturbed since establishment, making the location ideal to study mechanisms underlying invasion processes (Hulme et al., 2014). Potential confounding factors were largely considered by inclusion of altitude in the analyses. Altitude is an important factor shaping the environment in Amani, and there is a steep altitudinal gradient within the botanical garden. Altitude in ABG primarily affects rainfall and soil conditions and our manuscript describes how altitude is related to many soil variables. However, apart from organic matter content, the differences in soil chemistry or the effect of altitude had no significant effect on germination in the Petri dish study and altitude was also unrelated to the results of Study 3, which seems to confirm that the alien tree species were the main driver of the observed differences in germination and seedling recruitment.

One of the most striking findings of our study was the difference in the relationships between seedling abundance and species richness of alien and native species. The positive relationship between native seedling species richness and abundance appears typical for “normal” ecological systems: With increasing sample size, the number of detected species increases too (Gotelli & Colwell, 2001). The absence of a similar relationship in alien seedlings suggests that a different mechanism may be at play, which may reflect differences in, or a larger variety of dispersal strategies among alien species, and/or the more clumped occurrence of alien trees in ABG as compared to native species.

The higher average number of seedlings per alien than per native species may indicate that alien species produce more seeds than native species (Pysek & Richardson, 2007) or that alien species become more easily established, but the selection of the alien plant species in our study and their locations did not take the surrounding environment into account. For example, differences in native versus alien seedling numbers may also be due to enemy release, resulting in higher seedling survival and establishment of alien species than of native species (Colautti, Ricciardi, Grigorovich, & MacIsaac, 2004; DeWalt, Denslow, & Ickes, 2004), and the presence and abundance of alien species in the direct surroundings of the plots.

Dawson et al. (2009) studied factors explaining the extent of spread of alien plant species in the AGB and found that it was related to a number of factors, including the mode of spread and the number of seeds produced. These factors do not, however, necessarily lead to impact. For example, the number of native seedlings and species recorded under the widespread *Psidium guajava* were among the lowest recorded, and while the number of native species recorded under *Elaeis guineensis* was close to the average of the native species recorded under all 25 alien species, the number of native seedlings was among the lowest. By contrast, the number of native seedlings recorded under *Maesopsis eminii* was close to the average across alien species and the native species diversity was twice the average number. The comparison of the impact of these alien tree species illustrates the diversity of impacts of widespread alien tree species in the ABG and corroborates Kueffer and Daehler's (2009) findings that some alien tree species may have beneficial effects on the recruitment of native plant species. *Maesopsis eminii* was planted as nursery tree for endemic species, but it has since become one of the most widespread alien tree species in Amani, and it has been suggested to have a negative impact on native biodiversity as a result of its competitiveness (Binggeli & Hamilton, 1993). Our results do not provide strong evidence for different effects of *M. eminnii* on recruitment of native species of early and late successional stages, but the number of early successional seedlings and species was somewhat lower than the number of climax seedlings and species (Binggeli & Hamilton, 1993; Hulme, Burslem, et al., 2013). The reported effects on soil chemistry and the different rooting morphology and lack of mycorrhizae in *Maesopsis*‐dominated forest (Binggeli & Hamilton, 1993) may affect the ability of the seedlings to reach maturity. However, our data do not provide indications for the survival and future development of the recorded native seedlings. Further research is needed to assess the impact of alien species on forest composition and succession.

The three alien tree species highlighted above represent rapidly spreading alien species with different degrees of impact. For management that aims at minimizing the impacts of alien plants, predictors of impact would help the targeting of the potentially most harmful species. Species that have a limited spatial distribution in the exotic range are unlikely to have widespread impacts and the impact of widespread species that per unit area have limited impacts are equally unlikely to seriously affect native biodiversity. Hence, more multispecies studies that assess patterns in impacts are needed, because management should target alien species, such as *Psidium guajava*, that both spread rapidly and cause major impact on native species, biodiversity, and ecosystem functioning.

## CONFLICTS OF INTEREST

The authors declare that there is no conflicts of interests.

## AUTHOR CONTRIBUTION

Samson Aman Samson Kiswaga: Conceptualization (equal); Formal analysis (equal); Investigation (lead); Methodology (equal); Writing‐original draft (lead); Writing‐review & editing (equal). John Richard Mbwambo: Conceptualization (equal); Methodology (equal); Resources (equal); Supervision (equal); Writing‐original draft (equal); Writing‐review & editing (equal). Deo Shirima: Resources (equal); Supervision (equal); Writing‐review & editing (equal). Ahmed Mndolwa: Conceptualization (equal); Investigation (equal); Writing‐review & editing (equal). Urs Schaffner: Conceptualization (equal); Funding acquisition (lead); Project administration (lead); Writing‐review & editing (supporting). René Eschen: Conceptualization (equal); Data curation (equal); Formal analysis (equal); Methodology (equal); Supervision (equal); Visualization (lead); Writing‐original draft (supporting); Writing‐review & editing (lead). 

## Supporting information

Appendix S1‐S4

## Data Availability

Data are available through Dryad: https://doi.org/10.5061/dryad.51c59zw4x.
