# Supplementary material for: More widespread alien tree species do not have larger impacts on regeneration of native tree species in a tropical forest reserve
Source: Ecol Evol. 2020 Apr 12;10(11):5034–44. doi: 10.1002/ece3.6256 (PMC7297787; doi:10.1002/ece3.6256)
Supplement: Supplementary file 1 — Appendix S1‐S4 [file ECE3-10-5034-s001.docx]

### Appendices

**Appendix 1.** Plant species used to assess the effect of selected native and alien plant species on germination and growth of native trees in the Amani Botanical Garden. The Table indicates the species name, family, whether the species is native or alien, the extent of spread of the alien species (the number of plots in the Amani Botanical Garden where each alien species has been found in addition to its original planting location), the altitude of the location of the plant species where the soil was collected (m.a.s.l.) and where the field studies were conducted, and the studies in which each species was included.

| Species | Family | Growth form | Native/alien | Extent of spread | Altitude | Study 1 | Study 2 | Study 3 |
| --- | --- | --- | --- | --- | --- | --- | --- | --- |
| *Funtumia africana* (Benth.) Stapf | Apocynaceae | Tree | Native | - | 470 | x | x | x |
| *Isoberlinia scheffleri* (Harms) Greenway | Leguminosae | Tree | Native | - | 934 | x | x | x |
| *Macaranga capensis* (Baill.) Sim | Euphorbiaceae | Tree | Native | - | 960 | x | x | x |
| *Acer oblongum* Wall. ex DC. | Sapindaceae | Tree | Alien | 0 | 1019 | - | x | x |
| *Albizia chinensis* (Osbeck) Merr. | Leguminosae | Tree | Alien | 4 | 453 | - | x | x |
| *Albizia saman* (Jacq.) Merr. | Leguminosae | Tree | Alien | 22 | 477 | x | x | x |
| *Arenga pinnata* (Wurmb) Merr. | Arecaceae | Palm | Alien | 21 | 477 | x | x | x |
| *Artocarpus heterophyllus* Lam. | Moraceae | Tree | Alien | 6 | 461 | - | x | x |
| *Carludovica palmata* Ruiz & Pav. | Cyclanthaceae | Tree | Alien | 4 | 885 | - | x | x |
| *Castilla elastica* Cerv. | Moraceae | Tree | Alien | 18 | 618 | - | x | x |
| *Cedrela odorata* L. | Meliaceae | Tree | Alien | 43 | 470 | - | x | x |
| *Cinnamomum camphora* (L.) J.Presl. | Lauraceae | Tree | Alien | 10 | 1014 | - | x | x |
| *Clidemia hirta* (L.) D. Don | Melastomataceae | Tree | Alien | 75 | 884 | - | x | x |
| *Coffea canephora* Pierre ex A.Froehner | Rubiaceae | Tree | Alien | 2 | 448 | - | x | x |
| *Cordia alliodora* (Ruiz & Pav.) Oken | Boraginaceae | Tree | Alien | 30 | 815 | x | x | x |
| *Cryptomeria japonica* (Thunb. ex L.f.) D.Don | Cupressaceae | Tree | Alien | 0 | 1096 | x | x | x |
| *Elaeis guineensis* Jacq. | Arecaceae | Palm | Alien | 39 | 571 | x | x | x |
| *Ficus elastica* Roxb. ex Hornem. | Moraceae | Tree | Alien | 0 | 879 | x | x | x |
| *Hevea brasiliensis* (Willd. ex A.Juss.) Müll.Arg. | Euphorbiaceae | Tree | Alien | 1 | 466 | - | x | x |
| *Hovenia dulcis* Thunb. | Rhamnaceae | Tree | Alien | 16 | 888 | x | x | x |
| *Hura crepitans* L. | Euphorbiaceae | Tree | Alien | 4 | 491 | x | x | x |
| *Lantana camara* L. | Verbenaceae | Tree | Alien | 39 | 891 | - | x | x |
| *Maesopsis eminii* Engl. | Rhamnaceae | Tree | Alien | 47 | 955 | x | x | x |
| *Myroxylon peruiferum* L.f. | Leguminosae | Tree | Alien | 0 | 629 | - | x | x |
| *Schizostachyum dullooa* (Gamble) R.B.Majumdar | Poaceae | Bamboo | Alien | 22 | 447 | - | x | x |
| *Phyllostachys aureosulcata* McClure | Poaceae | Bamboo | Alien | 0 | 505 | - | x | x |
| *Phyllostachys bambusoides* Siebold & Zucc | Poaceae | Bamboo | Alien | 15 | 604 | x | x | x |
| *Phytelephas macrocarpa* Ruiz & Pav. | Arecaceae | Palm | Alien | 3 | 455 | - | x | x |
| *Piper aduncum* L. | Piperaceae | Tree | Alien | 22 | 444 | - | x | x |
| *Psidium cattleianum* Afzel. ex. Sabine | Myrtaceae | Tree | Alien | 15 | 1071 | x | x | x |
| *Psidium guajava* L. | Myrtaceae | Tree | Alien | 38 | 860 | - | x | x |
| *Psidium guineense* Sw. | Myrtaceae | Tree | Alien | 2 | 808 | - | x | x |
| *Senna occidentalis* (L.) Link | Leguminosae | Tree | Alien | 2 | 488 | - | x | x |
| *Syzygium jambos* (L.) Alston | Myrtaceae | Tree | Alien | 35 | 455 | x | x | x |

**Appendix 2.** Characteristics of the soils taken under 12 alien and three native plant species in ABG, used in Study 1.

Table A1. Means ± standard errors for soil variables measured in samples taken under four replicate trees per species.

|  | N_tot_ (%) | C_Org_ (%) | Na (mg/kg) | K (mg/kg) | P_Bray_ (mg/kg) | Ca (mg/kg) | Mg (mg/kg) |
| --- | --- | --- | --- | --- | --- | --- | --- |
| *Arenga pinnata* | 0.2 ± 0.02 | 2.59 ± 0.11 | 0.17 ± 0.02 | 0.19 ± 0.02 | 1.16 ± 0.79 | 7.93 ± 1.38 | 2.27 ± 0.35 |
| *Albizia samuni* | 0.3 ± 0.03 | 3.20 ± 0.37 | 0.17 ± 0.03 | 0.24 ± 0.03 | 1.43 ± 0.83 | 5.88 ± 1.24 | 1.29 ± 0.25 |
| *Creptomeria japonica* | 0.34 ± 0.03 | 4.64 ± 0.38 | 0.26 ± 0.02 | 0.20 ± 0.02 | 0.30 ± 0.07 | 4.74 ± 1.29 | 1.54 ± 0.34 |
| *Cordia alliodora* | 0.24 ± 0.02 | 3.47 ± 0.26 | 0.20 ± 0.02 | 0.24 ± 0.02 | 0.98 ± 0.59 | 10.95 ± 1.29 | 3.16 ± 0.44 |
| *Elaeis guineensis* | 0.26 ± 0.02 | 3.19 ± 0.40 | 0.17 ± 0.03 | 0.42 ± 0.11 | 0.20 ± 0.03 | 6.23 ± 0.50 | 2.60 ± 0.31 |
| *Funtumia africana* | 0.38 ± 0.01 | 4.45 ± 0.32 | 0.20 ± 0.02 | 0.34 ± 0.03 | 1.21 ± 0.37 | 15.76 ± 1.70 | 3.36 ± 0.27 |
| *Ficus elastica* | 0.29 ± 0.04 | 3.59 ± 0.40 | 0.21 ± 0.02 | 0.22 ± 0.03 | 0.30 ± 0.12 | 5.72 ± 1.37 | 2.05 ± 0.26 |
| *Hura crepitans* | 0.33 ± 0.03 | 3.77 ± 0.49 | 0.31 ± 0.05 | 0.72 ± 0.22 | 1.73 ± 0.78 | 11.48 ± 1.44 | 4.95 ± 1.33 |
| *Homis dulis* | 0.31 ± 0.03 | 4.41 ± 0.51 | 0.23 ± 0.03 | 0.21 ± 0.01 | 0.24 ± 0.07 | 7.51 ± 2.03 | 2.83 ± 0.60 |
| *Isobellinia scheffleri* | 0.49 ± 0.10 | 5.90 ± 0.50 | 0.25 ± 0.04 | 0.33 ± 0.07 | 2.19 ± 0.77 | 3.21 ± 0.40 | 0.92 ± 0.09 |
| *Macaranga capensis* | 0.25 ± 0.04 | 3.57 ± 0.46 | 0.22 ± 0.03 | 0.15 ± 0.01 | 0.32 ± 0.10 | 4.44 ± 0.68 | 1.39 ± 0.47 |
| *Maesopsis emenii* | 0.37 ± 0.03 | 5.49 ± 0.46 | 0.29 ± 0.04 | 0.21 ± 0.02 | 0.77 ± 0.23 | 5.03 ± 2.04 | 2.98 ± 1.76 |
| *Phyllostachys bambusoides* | 0.26 ± 0.03 | 3.13 ± 0.20 | 0.20 ± 0.03 | 0.30 ± 0.03 | 0.14 ± 0.01 | 13.78 ± 1.47 | 4.16 ± 0.05 |
| *Psidium cattleianum* | 0.25 ± 0.02 | 3.42 ± 0.22 | 0.23 ± 0.03 | 0.09 ± 0.01 | 0.17 ± 0.04 | 3.52 ± 0.95 | 0.86 ± 0.20 |
| *Syzigium jambos* | 0.24 ± 0.03 | 3.22 ± 0.46 | 0.21 ± 0.02 | 0.19 ± 0.03 | 0.41 ± 0.15 | 4.43 ± 0.60 | 2.19 ± 0.50 |

|  | pH_H2O_ | EC (me/100g) | CEC (me/100g) | % Sand | % Coarse silt | % Fine silt | % Clay |
| --- | --- | --- | --- | --- | --- | --- | --- |
| *Arenga pinnata* | 5.50 ± 0.29 | 0.14 ± 0.02 | 15.97 ± 1.73 | 55.20 ± 5.08 | 6.80 ± 0.80 | 11.00 ± 2.00 | 27.00 ± 4.22 |
| *Albizia samuni* | 4.94 ± 0.32 | 0.19 ± 0.04 | 13.18 ± 2.23 | 52.00 ± 3.63 | 3.20 ± 0.49 | 14.80 ± 0.49 | 30.00 ± 3.41 |
| *Creptomeria japonica* | 4.92 ± 0.13 | 0.16 ± 0.01 | 11.44 ± 2.41 | 49.20 ± 3.20 | 2.80 ± 0.80 | 13.60 ± 0.75 | 34.40 ± 3.06 |
| *Cordia alliodora* | 5.94 ± 0.04 | 0.20 ± 0.02 | 21.90 ± 3.56 | 46.60 ± 3.97 | 4.60 ± 1.08 | 16.20 ± 1.28 | 32.60 ± 3.54 |
| *Elaeis guineensis* | 5.92 ± 0.12 | 0.17 ± 0.02 | 13.73 ± 1.55 | 52.00 ± 3.74 | 4.40 ± 1.17 | 15.20 ± 0.80 | 28.40 ± 3.92 |
| *Funtumia africana* | 6.06 ± 0.05 | 0.22 ± 0.01 | 26.64 ± 1.98 | 55.60 ± 3.31 | 5.60 ± 0.75 | 14.00 ± 2.83 | 24.80 ± 3.14 |
| *Ficus elastica* | 5.17 ± 0.21 | 0.19 ± 0.05 | 18.25 ± 2.55 | 40.00 ± 4.69 | 9.50 ± 5.56 | 17.50 ± 1.26 | 33.00 ± 3.70 |
| *Hura crepitans* | 6.30 ± 0.16 | 0.37 ± 0.08 | 24.27 ± 3.49 | 54.80 ± 2.73 | 6.00 ± 0.63 | 13.60 ± 0.98 | 25.60 ± 1.94 |
| *Homis dulis* | 5.58 ± 0.20 | 0.17 ± 0.03 | 16.89 ± 3.60 | 38.50 ± 2.63 | 3.00 ± 0.58 | 17.00 ± 1.00 | 41.50 ± 2.50 |
| *Isobellinia scheffleri* | 4.30 ± 0.40 | 0.21 ± 0.03 | 9.40 ± 0.67 | 49.33 ± 4.37 | 5.33 ± 3.33 | 14.00 ± 2.00 | 31.33 ± 7.06 |
| *Macaranga capensis* | 5.30 ± 0.22 | 0.15 ± 0.04 | 10.18 ± 1.40 | 56.80 ± 2.42 | 3.60 ± 0.40 | 13.60 ± 1.17 | 26.00 ± 2.28 |
| *Maesopsis emenii* | 4.58 ± 0.29 | 0.16 ± 0.02 | 14.90 ± 6.32 | 53.00 ± 4.47 | 2.00 ± 0.00 | 10.00 ± 1.26 | 35.00 ± 3.31 |
| *Phyllostachys bambusoides* | 6.20 ± 0.10 | 0.20 ± 0.01 | 25.16 ± 2.01 | 51.00 ± 5.97 | 4.80 ± 0.80 | 15.40 ± 1.99 | 28.80 ± 4.18 |
| *Psidium cattleianum* | 5.24 ± 0.20 | 0.10 ± 0.00 | 7.70 ± 1.38 | 58.40 ± 2.79 | 2.00 ± 0.00 | 10.00 ± 0.63 | 29.60 ± 2.40 |
| *Syzigium jambos* | 5.46 ± 0.21 | 0.12 ± 0.01 | 11.07 ± 1.33 | 54.40 ± 4.07 | 4.40 ± 1.47 | 12.80 ± 0.80 | 28.40 ± 4.53 |

Figure A1. Correlation coefficients for all combinations of soil characteristics and altitude. The colours and shape of the symbols indicate the strength and direction of the correlations.


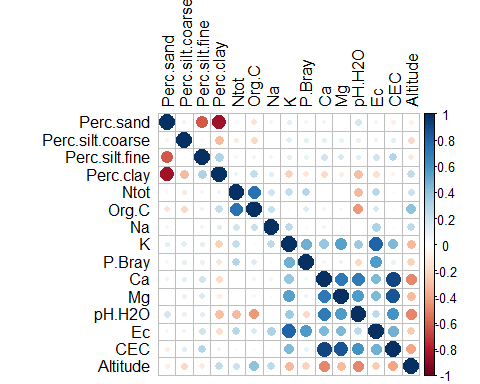


**Appendix 3.** Statistical model output.

Soil characteristics. Analysis of Deviance Table (Type II Wald chisquare tests).

Model: lmer(Response~Extent.of.spread+Altitude+(1|Species))

Response CEC Mg P.Bray log_10_(K) log_10_(Na) log_10_(C_org_)

df Chi.sq P Chi.sq P Chi.sq P Chi.sq P Chi.sq P Chi.sq P

Extent of spread 1 0.873 0.350 0.101 0.751 0.365 0.546 0.645 0.422 0.077 0.782 1.168 0.280

Altitude 1 2.833 0.092 1.731 0.188 4.471 0.034 4.381 0.036 2.638 0.104 10.269 0.001

Response log_10_(N_tot_) log_10_(pH_H2O_) %.clay %.sand %.silt_fine_ %.silt_coarse_

df Chi.sq P Chi.sq P Chi.sq P Chi.sq P Chi.sq P Chi.sq P

Extent of spread 1 0.017 0.895 0.978 0.323 0.814 0.367 0.204 0.651 3.683 0.055 0.970 0.325

Altitude 1 2.821 0.093 5.417 0.020 5.967 0.015 0.197 0.657 4.264 0.039 0.911 0.340

Study 1 - Petri dish experiment. Analysis of Deviance Table (Type II Wald chisquare tests)

Model: glmmPQL(Germinated.seeds.Funtumia~AC*( Extent.of.spread +Life.form+Alien.native),~1|Soil.origin,family=quasipoisson)

Response Germinated Funtumia seeds

df Chi.sq P

Activated carbon 1 0.061 0.805

Extent of spread 1 1.419 0.234

Life form 2 0.004 0.998

Alien/native 1 2.091 0.148

Activated carbon x Extent of spread 1 0.122 0.727

Activated carbon x Life form 2 2.741 0.254

Activated carbon x Alien/native 1 1.025 0.311

Model: glmmPQL(Germinated.seeds.Funtumia~Org.C,~1|Soil.origin,family=quasipoisson)

Response Germinated Funtumia seeds

df Chi.sq P

Organic carbon 1 8.853 0.003

Study 2 – Germination and survival in the field. Analysis of Deviance Table (Type II Wald chisquare tests)

Model: glm(Germinated~ Extent.of.spread *Native+Native*Alien.native+ Extent.of.spread *Native*Life.form,family=quasipoisson,na.action = na.omit)

Response Germinated seeds

df Chi.sq P

Extent of spread 1 0.515 0.473

Native tree species 2 29.415 <0.001

Alien/native 1 0.607 0.436

Life.form 2 9.330 0.009

Extent of spread x Native tree species 2 1.465 0.481

Alien/native x Native tree species 2 0.742 0.690

Extent of spread x Life form 2 4.782 0.092

Model: glm(Survived~Dawson.number*Native+Native*Alien.native+ Extent.of.spread *Life.form,family=quasipoisson,na.action = na.omit)

Response Surviving seedlings

df Chi.sq P

Extent of spread 1 0.432 0.511

Native tree species 2 27.218 <0.001

Alien/native 1 0.002 0.967

Life.form 2 4.103 0.129

Extent of spread x Native tree species 2 1.047 0.593

Alien/native x Native tree species 2 0.358 0.836

Extent of spread x Life form 2 1.967 0.374

Study 3 – Recruitment under alien plant species in ABG.

Model: glmmPQL(Response ~Altitude*Status+ Extent.of.spread +Status*(Alien.native+Life.form),~1|Host.species,family=quasipoisson())

Response Species number

df Chi.sq P

Altitude 1 4.119 0.042

Successional stage 3 101.425 <0.001

Extent of spread 1 0.683 0.409

Alien/native 1 0.361 0.548

Life form 2 0.130 0.937

Altitude x Status 3 19.535 <0.001

Successional stage x Alien/native 3 1.683 0.641

Successional stage x Life form 6 3.928 0.686

Model: glmmPQL(Response~Altitude*Successional.stage+Extent.of.spread+Successional.stage *(Alien.native+ Life.form),~1|Host.species, family=quasipoisson())

Response Seedling number

df Chi.sq P

Altitude 1 0.215 0.643

Successional stage 3 183.916 <0.001

Extent of spread 1 0.184 0.668

Alien/native 1 0.183 0.669

Life form 2 2.787 0.248

Altitude x Status 3 2.803 0.423

Successional stage x Alien/native 3 22.002 <0.001

Successional stage x Life form 6 3.592 0.732

Model: aov(glm(Response~Altitude+Extent.of.spread*Alien.native+ Extent.of.spread *Life.form,family="quasipoisson"))

Response Total species richness

df Sum.Sq Mean.Sq F P

Altitude 1 3.61 3.61 2.36 0.139

Extent of spread 1 1.08 1.08 0.71 0.409

Alien/native 1 0.54 0.54 0.36 0.557

Life form 2 0.17 0.09 0.06 0.946

Extent of spread x Life form 1 0.05 0.05 0.04 0.853

Residuals 21 32.04 1.53

Response Native species richness

df Sum.Sq Mean.Sq F P

Altitude 1 1.13 1.13 1.34 0.260

Extent of spread 1 0.30 0.30 0.36 0.558

Alien/native 1 0.46 0.46 0.55 0.466

Life form 2 0.35 0.17 0.21 0.815

Extent of spread x Life form 1 0.32 0.32 0.38 0.545

Residuals 21 17.66 0.84

Response Alien species richness

df Sum.Sq Mean.Sq F P

Altitude 1 0.21 0.21 0.47 0.499

Extent of spread 1 0.03 0.03 0.07 0.799

Alien/native 1 0.05 0.05 0.11 0.746

Life form 2 0.33 0.16 0.37 0.697

Extent of spread x Life form 1 0.68 0.68 1.53 0.230

Residuals 21 9.29 0.44

Model: aov(glm(Response~Extent.of.spread*Life.form+Alien.native,family=gaussian())))

Response Litter depth

df Sum.Sq Mean.Sq F P

Extent of spread 1 14.58 14.58 1.160 0.293

Life form 2 23.81 11.90 0.947 0.403

Alien/native 1 37.73 37.73 3.002 0.097

Extent of spread x Life form 1 23.83 23.83 1.896 0.184

Residuals 22 276.57 12.57

Response Litter cover

df Sum.Sq Mean.Sq F P

Extent of spread 1 2 1.9 0.002 0.961

Life form 2 3330 1664.9 2.209 0.134

Alien/native 1 1958 1957.8 2.598 0.121

Extent of spread x Life form 1 1224 1224.0 1.624 0.216

Residuals 22 16581 753.7

**Appendix 4.** The criteria for assigning seedlings of native species to different successional stages.

- 1. **Successional stages according to growth characteristics and reproductive habit**

1. Pioneer (P) species

- Light tolerant
- Very fast growth and forest gaps colonizer
- Early mature (very short rotation < 10 yrs.)
- Produce light and abundance of seeds
- Long distance dissemination of seeds dispersed (birds, wind, bats)
- Regeneration strategy is soil seed bank
- Early age of first reproduction (1-5 yrs.)
- Seed dormancy is photo- or thermo-induced
- Low dependence on specific pollinators
- Very light wood structure
- Indicator of forest disturbance
- Indicator of secondary forest
- Invasive when introduce into new habit.

1. Secondary (Early/Late) (S) species

- Shade intolerant and tolerant in juvenile stage
- Fast to medium slow growth
- Intermediate or relative late rotation (Age 10 -100 yrs.)
- Medium, small to medium amount of seeds and fruits production
- Light to medium hard – wood structure
- Early or late forest gap recovery
- Indicator of Secondary forest association with pioneer species
- dissemination seeds- dispersed (Birds, bats or wind), long distances
- Seed dormancy is absent
- Regeneration strategy is seedlings bank
- Age at first reproduction are intermediate and relatively late (5, 10-20 yrs.)
- High dependence on specific pollinators

1. Climax (C) species

- Shade tolerant
- Slow or very slow growth – rate
- Late mature (very long rotation >100 yrs.)
- Produce large of heavy fruits and seeds
- Short distance dissemination of seeds dispersed (gravity, mammals, birds and water)
- Seed dormancy is innate (embryo immaturity)
- Regeneration strategy is seedlings bank
- Age of first reproduction (>20 yrs.)
- High dependence on specific pollinators
- Heavy and hard wood structure
- Indicator of high forest (Climax stage) in succession
- Mostly are emergent in forest canopy
- Indicator of primary forest (undisturbed)

**4.2 Successional stages of listed species in Study 3**

*Albizia adianthifolia* S

*Alchornea hirtella* P

*Allanblackia stuhlmannii* C

*Allophylus occidentalis* S

*Anisophyllea obtusifolia* C

*Annickia kummeriae* C

*Anthocleista grandiflora* S

*Antiaris toxicaria* P

*Bersama abyssinica* C

*Blighia unijugata* S

*Bombax rhodognaphalon* S

*Bridelia micrantha* P

*Cephalosphaera usambarensis* C

*Chassalia parvifolia* P

*Chrysophyllum perpulchrum* C

*Cola scheffleri* S

*Diospyros amaniensis* S

*Drypetes usambarica* C

*Englerodedron usambarense* C

*Funtumia africana* P

*Hallea rubrostipulata* C

*Harungana madagascariensis* P

*Isoberlinia scheffleri* C

*Lannea amaniensis* C

*Lecaniodiscus fraxinifolius* C

*Leptonychia usambarensis* P

*Licuala spinosa* P

*Maesa lanceolata* P

*Maranthes goetzeniana* C

*Mesogyne insignis* S

*Milicia excelsa* C

*Newtonia buchananii* C

*Ocimum suave* P

*Oxyanthus speciosus* S

*Parinari excelsa* C

*Parkia filcoidea* C

*Pavetta amaniensis* S

*Phytelephas macrocarpa* P

*Podocarpus usambarensis* C

*Polyscias fulva* P

*Rauvolfia caffra* C

*Rauvolfia mombasiana* S

*Rothmania manganjae* S

*Schefflerodendron usambarense* C

*Shirakiopsis elliptica* S

*Sorindeia madagascariensis* S

*Stachytarpheta jamaicensis* P

*Synsepalum cerasiferum* C

*Synsepalum msolo* C

*Syzygium guineense* C

*Terminalia sambesiaca* C

*Toona ciliata* P

*Trichilia emetica* C

*Trilepsium madagascariense* S

*Xymalos monospora* P

*Zanha golungensis* C
